# Improved Biopharmaceutical Performance of Coenzyme Q10 Through Solid Lipid Nanoparticles for Enhanced Brain Delivery

**DOI:** 10.1155/sci5/9034181

**Published:** 2025-06-16

**Authors:** Shimul Halder, Faria Nasrin, Manik Chandra Shill, Madhabi Lata Shuma, Md. Zakir Sultan, Md. Selim Reza

**Affiliations:** ^1^Department of Pharmaceutical Technology, Faculty of Pharmacy, University of Dhaka, Dhaka 1000, Bangladesh; ^2^School of Pharmacy, Brac University, KHA 224, Progati Sarani, Merul Badda, Dhaka 1212, Bangladesh; ^3^Department of Pharmaceutical Sciences, North South University, Dhaka 1229, Bangladesh; ^4^Department of Pharmacy, School of Pharmacy and Public Health, Independent University Bangladesh, Dhaka 1229, Bangladesh; ^5^Centre for Advanced Research in Sciences, University of Dhaka, Dhaka 1000, Bangladesh

**Keywords:** BBB, coenzyme Q10, solid lipid nanoparticles, solvent injection, systemic exposure

## Abstract

Coenzyme Q10 (CoQ) is a powerful antioxidant with neuroprotective characteristics; nevertheless, its clinical use is constrained by inadequate solubility, diminished bioavailability, and limited blood–brain barrier (BBB) penetration. Solid lipid nanoparticles (SLNs) offer a promising approach to improve the biopharmaceutical characteristics and targeted delivery of CoQ to the brain. This study focuses on the strategic formulation and optimization of SLN-CoQ to improve solubility, oral absorption, and BBB permeability. The SLNs with drug loading of 2.5% (w/w) were prepared using a solvent injection technique and physicochemically characterized employing encapsulation efficiency, drug loading, particle size, zeta potential, surface morphology, crystallinity, in vitro drug release behavior, and mucus penetrating behavior. Pharmacokinetic studies were conducted in rats (100 mg-CoQ/kg, p.o.) after oral administration to elucidate the possible enhancement in the oral absorption of CoQ. The SLN-CoQ (F2) exhibited favorable physicochemical characteristics, including optimal particle size (91.6 ± 8.2 nm), zeta potential (−41.7 ± 1.03 mV), high encapsulation efficiency (85.2 ± 5.0), distinct surface morphology, reduced crystallinity, enhanced drug release, and better mucus penetration than crystalline CoQ. In the dissolution test, SLN-CoQ demonstrated a significant enhancement in the dissolution profile of CoQ as exhibited by an 83.6-fold higher dissolved amount of CoQ in 120 min in water in the F2 formulation ratio. Moreover, in the artificial mucus test, a 42-fold increase in mucus permeation was observed for the F2 formulation compared to the crystalline drug. Orally administered CoQ exhibited a higher systemic exposure of CoQ (3.6-fold higher) in SLLN-CoQ compared to crystalline CoQ, with prolonged circulation time and improved tissue distribution (3-fold higher) in rats. The findings suggest that SLN-CoQ offers a feasible nanotechnological method for enhanced drug transport to the brain, potentially aiding therapeutic approaches for neurodegenerative diseases, including Parkinson's and Alzheimer's.

## 1. Introduction

A naturally occurring antioxidant, coenzyme Q10 (CoQ), sometimes called ubiquinone, is essential for synthesizing cellular energy and defense against oxidative stress [1]. It is a key component of the mitochondrial electron transport chain, which helps produce adenosine triphosphate (ATP) necessary for cellular processes [2]. Furthermore, CoQ has a wide range of pharmacological potential, such as neuroprotective, anti-inflammatory, and antiapoptotic actions [3, 4]. Owing to these characteristics, CoQ has been extensively studied for its potential as a treatment for a variety of illnesses, such as mitochondrial dysfunctions, neurodegenerative disorders (Parkinson's and Alzheimer's diseases), and cardiovascular diseases [2, 5, 6]. The dosing recommendation of CoQ varies with the indication and stage of pathogenesis. One clinical trial among 80 subjects in the early stage of Parkinson's disease showed that CoQ in the dose of 300–1200 mg/day given in four divided doses is well tolerated, whereas 1200 mg/day exhibited a significant benefit in neurodegenerative diseases compared to the control groups [7]. Another study suggested that 2400 mg/day should be considered as the dose was well tolerated among 17 subjects with Parkinson's disease. However, no conclusive decision was provided about the efficacy of the regimen [8]. Despite its promising therapeutic applications, the clinical use of CoQ has been significantly hindered by its poor bioavailability and limited ability to penetrate the blood–brain barrier (BBB) [9]. These limitations are primarily attributed to its hydrophobic nature, which results in poor solubility (4 ng/mL) in aqueous environments and reduced absorption in the gastrointestinal tract, ultimately impacting its therapeutic efficacy [10, 11]. Traditional oral CoQ formulations (such as soft gel capsules) often exhibit limited absorption, leading to suboptimal plasma concentrations and lower therapeutic efficacy [12]. CoQ's inability to cross the BBB further complicates its use in treating neurological diseases. The BBB is a selective permeability barrier, restricting the passage of many pharmacological agents, including CoQ, from the bloodstream into the central nervous system (CNS) [13, 14]. These pharmacokinetic limitations necessitate innovative drug delivery strategies to enhance CoQ transport across biological barriers while maintaining therapeutic efficacy.

Nanotechnology-based drug delivery methods, particularly polymeric nanocarriers such as nanoparticles, micelles, and hydrogels, have numerous benefits for administering poorly soluble bioactive molecules like CoQ [15–17]. These nanocarriers improve solubility, prolong circulation time, and improve bioavailability via tailored surface modifications and functionalization [18, 19]. Surface-engineered polymeric nanocarriers can be specifically constructed to target the BBB by receptor-mediated transcytosis or passive diffusion, thereby enhancing CoQ concentrations in the CNS [20, 21]. Recent breakthroughs in nanotechnology have revealed solid lipid nanoparticles (SLNs) as a novel approach to improve the bioavailability and targeted distribution of poorly soluble drugs [22, 23].

SLNs are submicron-sized, lipid-based colloidal carriers that provide numerous advantages compared to conventional drug delivery technologies [24]. The benefits encompass biocompatibility, biodegradability, simplicity of manufacturing, and the capacity to encapsulate both lipophilic and hydrophilic pharmaceuticals. Furthermore, SLNs protect sensitive molecules from degradation, prolong their release profiles, and enhance their stability. SLNs have attracted significant interest in transporting active pharmaceutical ingredients (APIs) across the BBB [25]. Their size, surface characteristics, and capacity to be altered with targeted ligands to enhance their interaction with particular receptors or transport processes at the BBB are the leading causes [26].

Additionally, SLNs are recognized for their ability to boost cellular absorption, enhancing encapsulated drugs' pharmacological efficacy [27]. SLNs are considered superior to liposomes because of the possible low encapsulation, drug leakage, and oxidation of the CoQ molecules encapsulated in liposomes [28, 29]. SLNs are made of solid lipids, whereas liposomes are vesicular structures composed of a phospholipid bilayer, contributing to their fluidic nature and drug-leeching tendency [23]. In a study by Gokce et al., a smaller nanoparticle size and a 15% higher CoQ encapsulation were observed in SLN compared to those in liposomes [30]. Nanocrystals being thermodynamically unstable and lacking a protective lipid layer are also excluded from potential CoQ nanoformulations [31]. By incorporating CoQ into SLNs, it is possible to overcome the challenges of its poor solubility and limited brain penetration, thereby potentiating the treatment of neurodegenerative diseases explicitly targeting brain delivery. However, little is currently known about the feasibility of this approach.

This study aims to elucidate the advancements in SLN systems for CoQ delivery, highlighting their impact on bioavailability enhancement, BBB transport mechanisms, and potential clinical applications. The discussion will encompass various SLNs, their physicochemical properties, and functionalization strategies to optimize CoQ delivery for superior therapeutic outcomes in CNS-related disorders.

## 2. Materials and Methods

### 2.1. Materials

Reference CoQ was procured from Sigma Aldrich, USA. NIPRO JMI Pharma, Dhaka, Bangladesh, generously provided working samples of CoQ. Beta-carotene was purchased from Sigma Aldrich, USA. Soy lecithin was sourced from Merck (Germany). BASF, Bangladesh, kindly provided D-alpha tocopheryl polyethylene glycol 1000 Succinate (TPGS). Organic solvents, including ethanol and tertiary butyl alcohol (TBA), were of analytical grade. Artificial mucus (AM) components were procured from Sigma Aldrich, USA. All other chemicals and reagents utilized were of high purity and used without modification.

### 2.2. Standard Curve Preparation and CoQ Content Analysis

A 100-mL CoQ stock solution was prepared in a volumetric flask by mixing 10 mg of CoQ in 25 mL of TBA and then diluting it with 100 mL of distilled water to get a final concentration of 100 μg/mL of CoQ. Serial dilution with a TBA and water mixture (TBA:water = 1:3) yielded working solutions with concentrations ranging from 5 to 25 μg/mL from the solutions above. A UV–visible spectrophotometer (EMC-61PCUV, EMCLAB, Germany) measured the working solutions' absorbance at 275 nm. A standard curve for CoQ was subsequently generated by plotting the mean absorbance value of each experiment at each concentration against the corresponding drug concentration. The equation derived from the standard curve was used to quantify the concentration of CoQ. Each measurement was done in triplicate.

### 2.3. Preparation of SLN-CoQ

CoQ, when administered with a meal, gets solubilized with the lipid content of the meal with the help of bile salt and absorbed by simple passive facilitated diffusion [32]. In this study, a similar condition of lipid-induced CoQ absorption was tried to materialize. Stearic acid and soya lecithin were used as lipids in SLN-CoQ. This will stimulate bile secretion in the intestine after oral intake and subsequent micellar solubilization of CoQ and fatty acid and improve uptake by enterocytes. The rationale behind using both solid and liquid lipids was to reduce the recrystallization tendency of the lipid molecules and the subsequent expulsion of encapsulated drug molecules [33]. Stearic acid was used as the skeleton lipid as the melting point of stearic acid is higher (68°C compared to CoQ (48°C), which will trap the molecules. Also, lipids with higher melting point tend to recrystallize rapidly, which inspired the investigators to exclude other lipids with high melting point [34]. Moreover, stearic acid, a simple fatty acid, needs no enzymatic processing by intestinal lipase, resulting in quicker micelle formation, which might reduce drug precipitation by faster CoQ incorporation in the micelle. TPGS, a water-soluble version of vitamin E, was employed as a surfactant because of its ability to fluidize the phospholipid bilayer, suppress drug efflux, and enhance the hydrophilicity of mixed micelles, contributing to better drug uptake [35, 36]. Moreover, TPGS reduces oxidative stress and causes the upregulation of uptake receptors in the BBB, enhancing CoQ concentration in brain tissues [4].

A solvent injection method was applied to prepare SLN-CoQ per the previous report [37]. In brief, the organic phase was prepared by adding a predetermined amount of CoQ (2.5% w/w), (MS7-H550-Pro DLAB, USA) stearic acid, and soy lecithin to 10 mL of TBA in a 50-mL beaker. The organic phase was continuously stirred at 37°C and 1500 rpm using a magnetic stirrer. A specified quantity of TPGS was dissolved in 50 mL of distilled water at 37°C and 1500 rpm using a magnetic stirrer to prepare the aqueous phase. Both of the phases were maintained at 37°C. The aqueous phase was placed in an overhead stirrer (WiseStir HS30D, DAIHAN Scientific, South Korea) and subjected to rotation at 1000 rpm. The organic phase was constantly injected into the aqueous phase with a 5-mL syringe at a 0.5-mL/sec rate. The dispersion had been stirred for 2 h and subjected to sonication for 15 min at 37°C. The acquired dispersion was subsequently put onto Petri dishes in thin layers and subjected to an oven (WTC Binder, model FD 56) at 37°C for 24 h, as higher dryer temperature would melt the lipid and cause leakage of the drug. The nanoparticle formulations were dried by forced convection method inside the oven, ensuring a homogeneous temperature control. The desiccated product was gathered with a spatula and preserved in glass vials at 4°C in a refrigerator. For selecting the suitable ratio of the excipients used in SLN, the prepared SLN-CoQ formulations were kept standing for 1 h to observe any creaming or sedimentation tendency indicative of the formulation instability. The formulations where no visible signs of phase separation were observed were considered for evaluations, such as drug entrapment, drug loading (%DL), and dissolution studies. Among all the formulations, the best six were chosen for further evaluation.

### 2.4. Entrapment Efficiency (%EE)

The %EE of SLN-CoQ was assessed by extracting the free drug (unencapsulated) from the SLN-CoQ using ultracentrifugation. A predetermined quantity of SLN-CoQ dispersion was centrifuged for 30 min at 12,000 rpm, which effectively pelleted the SLN while leaving the free drug in the supernatant. The supernatant was carefully collected and quantified using a UV–vis spectrophotometry at 275 nm to determine the unencapsulated or free CoQ. Using the formula, the %EE was determined:

(1) $$\begin{array}{c} \%\text{EE}=\left[\frac{\left(\text{Total CoQ }–\text{ Free CoQ}\right)}{\text{ Total CoQ}}\;\right]\times 100. \end{array}$$

### 2.5. Drug Loading

The %DL of SLN-CoQ was measured by assessing the quantity of CoQ contained within the SLNs concerning the total SLN formulation. An aliquot of the SLN dispersion was centrifuged at 12,000 rpm for 30 min to isolate the nanoparticles from the unbound drug. The supernatant has been analyzed for unencapsulated CoQ using a UV–vis spectrophotometry at 275 nm. Utilizing the following equation, the %DL was determined:

(2) $$\begin{array}{c} \%\text{DL}=\left(\frac{\text{Amount of CoQ in SLNs}}{\text{Total amount of SLNs}}\;\right)\times 100. \end{array}$$

### 2.6. In Vitro Dissolution Studies

An in vitro dissolution test was conducted in distilled water (pH 5.5) for 120 min using a magnetic stirrer (MS7-H550-Pro DLAB, USA) at a speed of 75 rpm and a temperature of 37 ± 0.05°C. The methodology was adapted from previously published studies that have evaluated the dissolution behavior of poorly water-soluble compounds like CoQ using similar in vitro conditions [38]. The dissolution test was done in 50-mL distilled water as one of the study's aims was to improve CoQ's aqueous solubility. Distilled water was chosen as the dissolution medium for its simplicity and reproducibility, especially when comparing formulations in a standardized environment without the influence of surfactants or complex biological fluids. While more complex media like simulated gastric or intestinal fluids could be used in later stages, the goal at this stage was to highlight the solubility-enhancing potential of the SLN system in a baseline aqueous environment. Additionally, using a magnetic stirrer instead of a conventional USP dissolution apparatus was driven by the small volume and limited sample size, which allowed us to work more efficiently and reduce material use. Crystalline CoQ, SLN-CoQ formulations, and a standard market product (UbiCare, 60-mg capsule, Popular Pharmaceuticals Ltd., Bangladesh) were all carefully weighed to keep the amount of CoQ in the 50-mL beaker at 5 mg. At 5, 15, 30, 45, 60, 75, 90, and 120 min, a 300-μL aliquot was withdrawn using a micropipette and replaced with an equivalent fresh dissolution medium. Each sample was diluted with a 1:3 solution of TBA and water after being centrifuged at 5000 × g for 30 min. Using a UV spectrophotometer, CoQ concentrations were measured in samples at 275 nm. All experiments were performed in triplicate.

### 2.7. AM Diffusion Test

According to the reported procedure, the mucus-penetrating behavior of SLN-CoQ was evaluated using an AM model [39]. A 0.28% (w/v) agarose solution was prepared to facilitate an assessment before the investigation. Agarose was solubilized in heated distilled water to create an agarose solution. The formulation of AM consisted of 0.295 mg of diethylenetriaminepentaacetic acid, 250 mg of porcine gastric mucin, 1 mL of RPMI-1640 medium, 250 μL of egg yolk emulsion, 250 mg of NaCl, and 110 mg of KCl in 25 mL of distilled water. All raw materials were added to 50 mL of distilled water in a volumetric flask and allowed to equilibrate for 2 h at 37°C. To check the penetration of CoQ to the BBB, we have kept the pH of 7 and a viscosity of 1 mPa·s of the prepared AM measured at 37°C, which are within the physiological range of brain mucus environments. One milliliter of agarose solution was introduced into a 5-mL microtube to create a lower gel layer. After gelation, 1 mL of AM was introduced to the microtube, followed by the precise addition of 300 μL of AM-dispersed CoQ samples atop the aforementioned layer. The microtubes were subsequently incubated for 30 min at 37°C. The upper AM layer was removed postincubation, and the residual agarose gel was liquefied at 60°C following three rinses with distilled water, after which CoQ was extracted twice using TBA. The entire system was thereafter transferred to a centrifuge tube and subjected to centrifugation at 5000 rpm for 10 min. 400 μL of supernatant was extracted and diluted with a 4-mL mixture of TBA and water in a 1:3 ratio. The absorbance was measured using a UV–visible spectrophotometer at 275 nm to ascertain the concentration of CoQ that traversed the AM layer. Each SLN sample was subjected to triplicate experimentation.

### 2.8. Particle Size Distribution

Using dynamic light scattering (DLS) with a Nanoparticle Analyzer (Nanopartica SZ100V, Horiba, Japan), the particle size, polydispersity index (PDI), and zeta potential of SLN-CoQ were determined. Prior to the measurement, the CoQ concentration was kept constant at 10 μg/mL. To prevent additional scattering effects, the SLN dispersion was diluted with deionized water. At a detection angle of 90°, measurements were carried out at 25°C. The average particle size and PDI were recorded to assess uniformity, while the zeta potential was measured to evaluate colloidal stability. Every measurement was performed three times.

### 2.9. Scanning Electron Microscopy (SEM)

The surface morphologies of crystalline CoQ, physical mixture, and SLN-CoQ were analyzed utilizing SEM (JEOL JSM-7610F, Japan). A minimal volume of SLN dispersion was drop-cast onto a carbon-coated copper grid and vacuum-dried. The dried samples were sputter-coated with a thin layer of platinum to improve conductivity and later examined using SEM at an accelerating voltage of 5–15 kV. The images were analyzed to assess particle shape, surface roughness, and aggregation behavior. Representative micrographs were obtained at various magnifications to confirm the homogeneity and structural integrity of the nanoparticles.

### 2.10. Differential Scanning Calorimetry (DSC)

DSC was conducted to assess the thermal properties and crystallinity of SLN-CoQ. Measurements were performed with a DSC analyzer (Netzsch, Germany). Samples comprising crystalline CoQ, physical mixtures, and SLN-CoQ were weighed (3 mg) and encapsulated in aluminum pans. In a nitrogen environment (50 mL/min), thermograms were generated at a heating rate of 10°C/min over a temperature range of 25°C–350°C. The melting temperatures, enthalpy, and peak shifts were examined to evaluate potential drug–lipid interactions and the amorphous or crystalline characteristics of the formulation.

### 2.11. X-Ray Powder Diffraction (XRPD)

XRPD was performed to examine the crystalline properties of CoQ within SLN using an X-ray powder diffractometer (PW 3040-X'Pert PRO PANalytical, Philips, The Netherlands) with Cu-Kα radiation (*λ* = 1.6 Å). Samples comprising crystalline CoQ, physical mixtures, and SLN-CoQ were examined throughout a 2*θ* range of 5°–35° at a scanning rate of 4°/min and a step size of 0.02°. Diffractograms were analyzed to determine differences in peak intensity and sharpness, indicating potential transitions to an amorphous state in the nanoparticle formulation.

### 2.12. Drug–Excipient Interaction

Fourier-transform infrared spectroscopy with attenuated total reflectance (FTIR-ATR) was employed to assess possible interactions between CoQ and the lipid matrix in SLN. Spectra were recorded using an FTIR spectrometer (Perkin Elmer, L160000A, USA) equipped with an ATR module. Samples, including crystalline CoQ, lipid excipients, physical mixtures, and SLN-CoQ, were scanned over the 4000–650 cm^−1^ at a resolution of 4 cm^−1^ with 32 accumulations per scan. Typical peaks were analyzed to identify potential shifts or alterations, indicating molecular interactions or structural modifications in the formulation. Each sample's baseline was adjusted and normalized during the study. To smooth the accumulated spectra, a nine-point smoothing function was applied.

### 2.13. Physicochemical Stability Study Under Accelerated Conditions

For the evaluation of physicochemical stability, about 10 mg of each CoQ sample was poured into a 5-mL brown-colored glass bottle under accelerated conditions in accordance with ICH Q1A(R2) guidelines [40]. The samples were stored at 40 ± 2°C or 40 ± 2°C/75 ± 0.5% relative humidity (RH) for 4 weeks and 60 ± 2°C for 2 weeks, respectively, in a stability chamber (Labcare Pvt. Ltd., Mumbai, India). After storage, the physicochemical characteristics of aged samples were evaluated in terms of physical appearance and potency.

### 2.14. Animals

Male Wistar rats, 8–9 weeks old and weighing between 250 and 300 g, were collected from North South University in Dhaka, Bangladesh. They were kept in cages in pairs. The rats were maintained under conventional laboratory settings (12 h dark/light cycle, 22 ± 2°C) with free access to food and water. Rats were randomly divided into two groups, and a total of *n* = 4 rats per group were used in the in vivo experiments. This sample size was determined based on preliminary data and a power analysis to detect statistically significant differences in the brain uptake of CoQ, considering ethical principles to minimize animal usage in accordance with the 3Rs (replacement, reduction, and refinement). Before the trial, the rats were acclimated for 1 week. Each animal study complies with the University of Dhaka's Faculty of Biological Sciences' Institutional Animal Care and Ethics Committee (Approval No. 235, August 30, 2023). The study also followed the guidelines set by the International Council for Laboratory Animal Science, the Nuffield Council on Bioethics, and the Council for International Organizations of Medical Sciences (CIOMS/ICLAS). For blinding the study, the samples (crystalline CoQ and SLN-CoQ) were prepared in identical containers and labeled with codes. The personnel involved in outcome assessment was blinded to the treatment allocation to reduce bias.

### 2.15. Pharmacokinetic Assessment

Rats were randomly divided into two groups, and a total of *n* = 4 rats per group were used in the in vivo experiments. To ascertain plasma concentrations of CoQ, rats received a single oral dose of crystalline CoQ (100 mg/kg) or SLN-CoQ (100 mg-CoQ/kg) suspended in 2 mL of distilled water, independent of the experimental group. Blood samples (0.5 mL) were collected from the tail vein at specified intervals (0.5, 1, 2, 4, 8, 12, 24, and 36 h postadministration) and centrifuged at 10,000 rpm for 10 min to extract the plasma, which was then stored at −20°C for subsequent analysis. The plasma samples were subsequently tested for CoQ content, utilizing a validated high-performance liquid chromatography (HPLC) reported previously [10]. The frozen samples were thawed at room temperature before examination. The subsequent procedure was implemented on the unfrozen plasma samples: Briefly, 0.1 mL of internal standard solution (beta-carotene, 5 μg/mL in methanol) was added to 0.25 mL of plasma in an Eppendorf tube. Then, 0.5 mL of methanol was added to the mixture for protein precipitation. Afterward, 0.75 mL of n-hexane was added. Following 5 min of vortexing, the mixture underwent centrifugation for 15 min at 5000 rpm. The transparent hexane layer was then moved to another tube, and an additional 0.75 mL of n-hexane was employed to replicate the extraction process. The plasma extracts were dried by evaporation under a nitrogen stream. Following dissolution in 1 mL of acetonitrile, the dry residue was fed into the HPLC system. The HPLC system comprised a reverse phase C18 column (4.6 × 250 mm, 5 μm) with a mobile phase of 60:40 v/v acetonitrile and isopropyl alcohol, flowing at a 1-mL/min rate. Calibration curves were established with standard CoQ solutions to determine plasma concentrations. Each rat's pharmacokinetic parameters were determined using noncompartmental analysis and the PK solver, an add-on application for Microsoft Excel [41].

### 2.16. Brain Tissue CoQ Levels

After blood samples were taken at the end of the experiment, rats were subjected to euthanasia with a single intraperitoneal dose of ketamine (300 mg/kg b.w.) to evaluate the distribution of SLN-CoQ in the brain tissue. After extraction, the rats' brains were cleansed in ice-cold saline, wiped using filter paper, and preserved for later examination. Later, the brain samples were thawed, carefully dissected, and homogenized in 5-mL saline and acetonitrile, weighing five times the weight of the tissue to facilitate protein precipitation. The homogenized samples were taken in Eppendorf and vortexed with n-hexane twice the volume of homogenized sample to isolate CoQ by liquid-liquid extraction. The extraction was done twice, and n-hexane samples following extraction were processed for CoQ quantification using an HPLC method similar to that used for plasma analysis.

### 2.17. Data Analysis

The graphs were generated using GraphPad Prism 8.0 (Graph Pad Software, La Jolla, CA). After doing a one-way ANOVA, Tukey's test for multiple comparisons was utilized to compare SLN-CoQ with crystalline CoQ statistically. Data expressed as mean ± standard deviation (S.D.) at *p* < 0.05 indicated statistical significance.

## 3. Results and Discussion

### 3.1. Optimization of SLN-CoQ Components

The SLN-CoQ formulations were prepared by varying the TPGS and soy lecithin amounts, keeping the fixed amount of CoQ and stearic acid among all formulations. A weak positive correlation was observed between the amount of soy lecithin and %EE, indicating a slight increase in drug entrapment with the rise of liquid lipid, probably due to increased flexibility and room for drug molecules in the lipid core [42]. On the other hand, a weak negative correlation was found between drug entrapment and TPGS, indicating a slight lowering of drug encapsulation with an increased amount of TPGS. The TPGS molecules might form a micelle at a higher surfactant concentration and trap the drug in a micelle core, contributing to low drug encapsulation. A similar reduction in drug entrapment was also observed in previous studies related to hydrophobic drug nanoparticle formulation [43–45].

**Figure 1:**
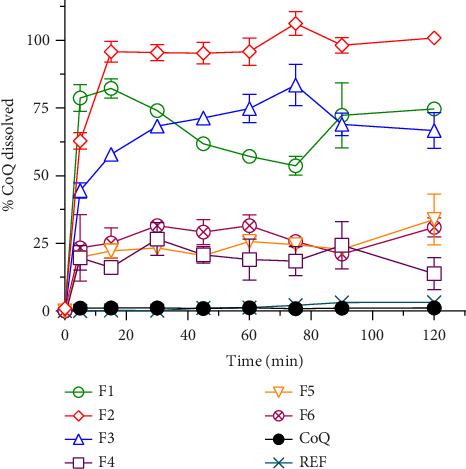
Dissolution tests of CoQ samples in water. ●, crystalline CoQ; ○, F1; ◇, F2; △, F3; □, F4; ▽, F5; ⊗, F6; and ×, ref. Data represent mean ± S.D. of three independent experiments.

The formulations of SLN-CoQ were optimized based on %EE, %DL, in vitro dissolution performance, and mucus penetrating properties to ensure an efficient drug delivery system for brain-targeted delivery. The %EE of SLN-CoQs ranged from 62.36% to 85.22%, and %DL ranged from 1.71% to 2.2%, where F2 showed the highest entrapment and loading (Table 1). This high %EE indicates the successful encapsulation of CoQ within the SLNs, minimizing drug loss during preparation and storage. The %DL, essential for ensuring therapeutic efficacy, was also optimized, providing an adequate amount of CoQ in each nanoparticle for effective drug delivery. This finding is similar to a previous study where increased surfactant concentration decreased %EE [46]. CoQ's solubility/dissolution behavior is also one of the most critical issues in therapeutic applications. In vitro dissolution assessments evaluate the drug release profile within the physiological environment and determine drug release behavior from various dosage forms. To determine whether various SLN-CoQ formulations can improve the dissolution behavior of CoQ in comparison to crystalline CoQ and the commercially available reference product, dissolution studies were conducted on all SLN-CoQ formulations in distilled water (Figure 1). The dissolution rate was observed by plotting the percentage of drug released against time for each sample (Figure 1). The initial drug release from crystalline CoQ was 1.1% at 15 min and remained unchanged at 30 min, indicating a limited dissolution behavior initially. At a time point of 120 min, the mean drug release was 1.1%, indicating that dissolution behavior did not improve significantly with time. The commercially available reference product exhibited a drug release of 0.21% at both the 15- and 30-min time points. It demonstrated a 3-fold increase in dissolution behavior compared to crystalline CoQ at 120 min. In contrast, the dissolution behaviors of CoQ from all SLN-CoQ formulations were significantly enhanced compared to those of crystalline CoQ and the reference commercial product. The improvement ranking was F2 > F1 > F3 > F6 > F4 > F5, with an 83.6 > 65.5 > 61.4 > 28 > 25.3 > 20.6-fold increase in % dissolution at 30 min compared to crystalline CoQ (Table 1). Based on the dissolution data, it was clear that all SLN-CoQ formulations exhibited burst drug release (Table 1).

Compared to conventional CoQ, formulations, such as oil-based soft gels and emulsions, have demonstrated only modest improvements in dissolution, typically ranging from 2- to 10-fold increases depending on the delivery system. For instance, self-emulsifying drug delivery systems (SEDDS) have reported up to a 6-fold improvement, while some polymeric nanoparticle systems have shown enhancements in the 10–20-fold range. In addition, in a study conducted by Piao et al., a 49 times increase in dissolution was observed from lipid nanoparticles compared to the coarse suspension of CoQ [47]. In another study of CoQ, solid dispersion dissolution in pH 6.8 phosphate buffer was found to be 85 times higher compared to the crystalline CoQ, and maximum drug release was achieved in 15 min in a burst release manner similar to our study, probably due to conversion of the drug in the amorphous form [48]. Furthermore, protein nanoparticles of CoQ showed much lower drug release, and only four times improvement was found compared to pure drug samples in PBS (pH = 7.4) dissolution media [49]. This enhanced dissolution behavior of the SLNs can be attributed to their smaller particle size and larger surface area and the lipid matrix's ability to improve drug solubilization and stability, which facilitates a more significant interaction with the dissolution medium and increases the solubility of CoQ. In this study, stearic acid serves as the solid lipid, providing a stable, hydrophobic matrix that forms the core of the nanoparticles, which primarily influences particle size by contributing to a rigid lipid structure and enhances EE by creating a solid matrix that effectively entraps CoQ. The soy lecithin, a liquid lipid and natural phospholipid, acts as an emulsifier and stabilizer that reduces interfacial tension, thereby helping to decrease the particle size and improve the homogeneity of the nanoparticles; it also enhances EE by promoting better lipid–drug compatibility and contributes a slight negative charge to the nanoparticle surface, influencing the zeta potential. TPGS stabilizes the nanoparticles by steric hindrance, reduces particle size, and improves EE by increasing drug solubility within the lipid matrix. Stearic acid tends to increase particle size individually due to its solid nature, while soy lecithin and TPGS reduce size through emulsification and surface stabilization. In combination, these excipients synergistically optimize particle size by balancing matrix rigidity and interfacial tension, enhance EE by improving lipid–drug compatibility and solubilization, and modulate zeta potential stearic acid being neutral, soy lecithin imparting slight negative charge, and TPGS contributing to steric stabilization rather than significant charge resulting in nanoparticles with improved colloidal stability and biopharmaceutical performance suitable for enhanced brain delivery.

The mucus penetration characteristics of the SLN-CoQ were assessed, as this is a crucial attribute for enhancing drug absorption through mucosal membranes and ensuring effective delivery to the brain [50]. The capacity of SLNs to traverse the mucus barrier is a pivotal element in improving the bioavailability of orally delivered formulations. In our work, mucus penetration was evaluated using an AM model that simulated the circumstances of the gastrointestinal mucosa. The findings indicated that SLN-CoQ markedly exceeded free CoQ regarding mucus penetration (Table 1). The quantities of CoQ in the agarose layer were observed in descending order as follows: 5.43, 3.88, 3.03, 2.72, 2.34, 1.77, and 0.13 μg/mL for F2, F4, F3, F6, F1, F5, and crystalline CoQ, respectively. The enhanced mucus-penetrating capability of SLNs is due to their small particle size, allowing for more efficient traversal through the dense mucus network compared to bigger particles [51]. Conversely, the SLN-CoQ was encapsulated with TPGS and lecithin. TPGS and lecithin are amphiphilic compounds, rendering the nanoparticle's outer portion hydrophilic. This may have minimized the hydrophobic interaction between lipids and CoQ with mucin, enhancing the mucus permeability of SLN-CoQ.

The surface properties of the SLNs, particularly the incorporation of surfactants, may facilitate improved interactions with mucus, enabling deeper penetration and extended retention in mucosal tissues [52]. The mucoadhesive characteristics of the SLNs improved their retention at the administration site, which is essential for enhancing drug absorption and bioavailability [53]. These findings indicate the application of SLN-CoQ as an efficacious formulation for improved delivery at the BBB, presenting a novel strategy for managing neurological diseases. Therefore, based on %EE, %DL, improved dissolution, and mucus penetrating properties, F2 was chosen as the most suitable one for extended physicochemical characterization and pharmacokinetic studies.

### 3.2. Physicochemical Characterization

**Figure 2:**
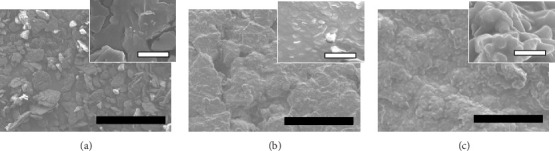
Microscopic images observed by scanning electron microscope. (a) Crystalline CoQ, (b) CoQ-physical mixture, and (c) SLN-CoQ. Each black and white bar represents 100 and 1 μm, respectively.

The physicochemical properties of SLN-CoQ were thoroughly characterized to assess their potential in improving biopharmaceutical performance and enhancing transport across the BBB. Particle size is critical in oral drug delivery systems, especially for crossing the BBB. DLS analysis revealed that the SLN-CoQ had an average particle size of 91.6 ± 8.2 nm with a PDI of 0.32 ± 0.02, indicating a homogeneous size distribution. The small size of these nanoparticles is essential for improving drug permeability across biological barriers like the GIT, as particles under 200 nm can more effectively penetrate these barriers [54]. The zeta potential measurements indicated a stable surface charge (−41.7 ± 1.03 mV), crucial for maintaining dispersion stability and preventing aggregation in physiological conditions. Additionally, when SLN-CoQ was kept at room temperature, no appreciable particle size or zeta potential changes were seen at least 3 h later, demonstrating the formulation's excellent colloidal stability. SEM imaging of the SLN-CoQ revealed smooth, spherical nanoparticles with consistent morphology and a well-defined surface, further confirming the results from DLS (Figure 2). The spherical shape enhances the potential for cellular uptake and improved interaction with mucus layers, facilitating better retention at the administration site and enhancing bioavailability. The well-defined surface morphology indicates the successful encapsulation of CoQ within the lipid matrix without significant aggregation, which is vital for maintaining the drug's therapeutic efficacy.

**Figure 3:**
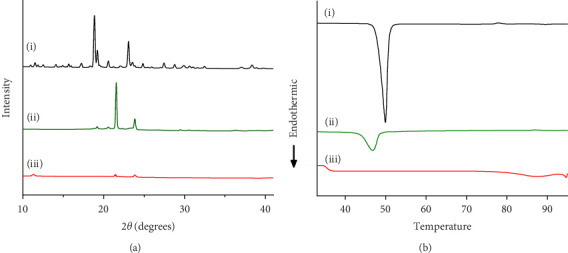
Crystallinity assessment of CoQ samples using (a) XRPD and (b) DSC. (i) Crystalline CoQ, (ii) CoQ-PM, and (iii) SLN-CoQ.

The crystallinity of CoQ in the SLN formulation was evaluated using XRPD, revealing a significant reduction in the crystalline structure of CoQ when encapsulated in the lipid matrix (Figure 3(a)). Crystalline CoQ displayed sharp, distinct crystalline peaks, indicating poor solubility while the SLN-CoQ exhibited less intense and fewer peaks, suggesting that the drug was present in a more amorphous state within the lipid matrix (Figure 3(a)). This transformation is advantageous as amorphous forms typically exhibit higher solubility, leading to faster dissolution rates [55]. DSC analysis further supported these findings, showing a broad and lowered melting point for CoQ in the SLNs compared to the sharp peak observed for the free drug (Figure 3(b)). This shift in the thermal behavior indicates that CoQ was successfully encapsulated in the lipid matrix, with the crystalline structure disrupted, enhancing its solubility and enabling sustained release. This thermal behavior alteration can also impact CoQ's release kinetics, as the amorphous drug form generally leads to faster dissolution and enhanced bioavailability compared to crystalline drug forms [55]. Additionally, the thermal stability of the SLNs was confirmed, with no significant changes in the lipid matrix upon heating, indicating that the formulation is stable under standard storage conditions. From prior studies, Onoue et al. demonstrated that nanoparticulate formulations can be more prone to degradation than their crystalline counterparts [56, 57]. To investigate the accelerated‐condition stability of our SLN‐CoQ samples, we conducted storage tests at 40°C for 4 weeks (dry), 40°C/75% RH for 4 weeks, and 60°C for 2 weeks and then examined key physicochemical attributes. After 4 weeks at 40°C and 2 weeks at 60°C, the SLN‐CoQ formulations exhibited excellent stability, with no noticeable changes in color or evidence of aggregation. In contrast, samples stored at 40°C/75% RH for 4 weeks developed large aggregates, likely due to moisture‐induced deliquescence. These findings indicate that the SLN matrix effectively maintains the amorphous state of CoQ under dry heat but requires humidity control to prevent water uptake. Overall, our data support the conclusion that SLNs can inhibit CoQ nucleation and crystal growth [58], thereby preserving its amorphous form, which underpins the enhanced dissolution and sustained oral absorption observed in our pharmacokinetic studies.

These physicochemical properties indicate that SLN-CoQ possesses the necessary characteristics for effective drug delivery, including improved dissolution, enhanced solubility, and controlled release. These are crucial for enhancing CoQ's bioavailability and ability to cross the BBB.

### 3.3. Drug–Polymer Interaction

**Figure 4:**
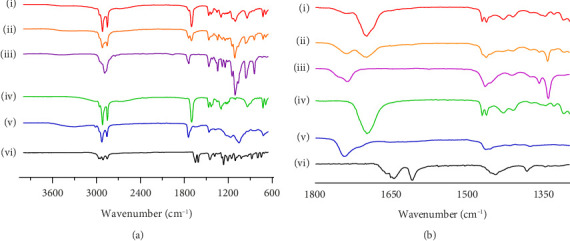
Drug–polymer interaction studies of CoQ samples using FT-IR. Baseline-corrected and normalized IRdata of CoQ samples in the spectral wavenumber region from (a) 4000 to 600 cm^−1^ and (b) 1800 to 1300 cm^−1^. (i) SL-CoQ, (ii) PM-CoQ, (iii) TPGS, (iv) SA, (v) soy lecithin, and (vi) crystalline CoQ.

FTIR-ATR analysis was employed to investigate the drug–polymer interactions in the formulation of SLN-CoQ, focusing on crystalline CoQ, SLN-CoQ, and key excipients such as TPGS, lecithin, stearic acid, and their physical mixtures. FTIR spectra of crystalline CoQ revealed characteristic peaks at 2964.26 cm^−1^ (C–H stretching), 1609.46 cm^−1^ (C=O stretching), and 1078.69 cm^−1^ (C–O stretching), which are typical of CoQ's molecular structure (Figure 4). In contrast, when CoQ was incorporated into SLNs, the FTIR spectrum of SLN-CoQ showed subtle shifts and alterations in the intensity of these peaks, suggesting interactions between CoQ and the lipid matrix components (Figure 4). Notably, the ester-linked vibrations of lecithin (phospholipid) at around 1742.51 cm^−1^ were observed to shift slightly, indicating a potential interaction with the CoQ molecule, likely through hydrophobic interactions or van der Waals forces, which could aid in the solubilization of CoQ within the lipid phase of SLNs [59]. Additionally, the peaks corresponding to stearic acid (C–H stretching at 2915.62 cm^−1^ and C=O stretching at 1698.16 cm^−1^) and TPGS (C–H bending at 1341.93 cm^−1^) also exhibited minor shifts upon incorporation into the SLN formulation (Figure 4). The alterations in the FTIR spectra of SLN-CoQ indicate the formation of a complex between the lipid excipients and CoQ, which may enhance encapsulation stability and solubility of CoQ within the lipid matrix. The physical mixture of CoQ with TPGS, lecithin, and stearic acid exhibited FTIR characteristics that were less distinct than those of SLN-CoQ, with no significant shifts or new peaks, suggesting negligible interactions between the drug and excipients in the unformulated condition (Figure 4). This reinforces the concept that lipid nanoparticles promote more robust interactions than simply a physical mixture, perhaps owing to the lipid core's improved structural integrity and interfacial characteristics in SLNs [60]. The inclusion of TPGS, a recognized solubilizer and surfactant, presumably improved bioavailability and aided the integration of CoQ into the SLNs, as indicated by the FTIR data [61]. These data might correlate with the improved bioavailability and brain tissue distribution of SLN-CoQ, as the interactions mediated by the excipient possibly facilitated superior encapsulation efficiency and regulated release properties. Thus, the FTIR-ATR results provide valuable insights into the molecular interactions between CoQ and its lipid-based carriers, affirming the role of SLNs in improving CoQ's stability, solubility, and overall bioavailability.

### 3.4. Pharmacokinetic Behavior of CoQ Samples

**Figure 5:**
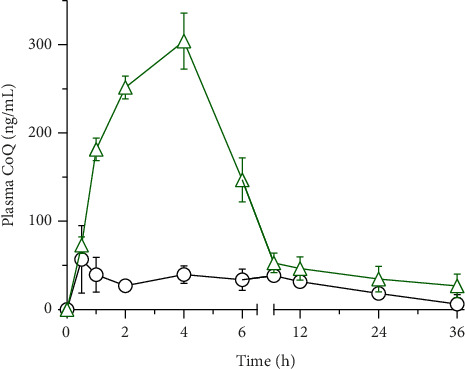
Plasma concentration–time profile of CoQ after the oral administration of CoQ samples in rats. ○, crystalline CoQ (100 mg/kg, p.o.), △, SLN-CoQ (100 mg-CoQ/kg, p.o.). Data represent mean ± S.E. of four experiments.

The pharmacokinetic behavior of CoQ formulations was assessed in rats following the oral administration of 100 mg/kg of crystalline CoQ and SLN-CoQ (Figure 5). The results demonstrated a marked improvement in the pharmacokinetic parameters of SLN-CoQ compared to that of crystalline CoQ. SLN-CoQ exhibited a significantly higher *C*_max_ (308.48 ± 49.25 ng/mL) than crystalline CoQ (69.15 ± 17.27 ng/mL), indicating that the SLN formulation enhances the peak plasma concentration of CoQ (Table 2). This improvement in *C*_max_ suggests better absorption, which could be attributed to the improved solubility exhibited by the SLN. Additionally, SLN-CoQ showed a delayed *T*_max_ (3.33 ± 1.15 h) compared to crystalline CoQ (1.67 ± 2.02 h), indicating a slower absorption rate, consistent with the controlled-release properties of SLNs. The increased AUC_0–6h_ (1002.5 ± 122.3 ng·h/mL) and AUC_0–∞_ (4203.75 ± 2189.11 ng·h/mL) for SLN-CoQ further reflect the enhanced bioavailability and sustained release of CoQ, providing prolonged drug exposure (Table 2). This contrasts with crystalline CoQ, which had lower AUC values, indicating that the SLN formulation extends the drug's existence in the systemic circulation. SLN-CoQ also demonstrated a longer MRT (35.27 ± 25.58 h) compared to crystalline CoQ (23.15 ± 9.49 h), suggesting that the drug stays in circulation for a longer period, further supporting the sustained-release behavior of the nanoparticles. Furthermore, SLN-CoQ showed a longer half-life (30.28 ± 20.64 h) compared to crystalline CoQ (15.34 ± 6.27 h), emphasizing the slow elimination rate of the nanoparticle formulation. Despite the slower elimination, the elimination rate constant (*K*_e_) for SLN-CoQ (0.046 ± 0.05 h^−1^) was similar to that of crystalline CoQ (0.049 ± 0.01 h^−1^), indicating that the overall drug clearance is slightly reduced but still within a favorable range for sustained therapeutic action.

The lipid nanoparticles probably enhanced lymphatic absorption, avoiding hepatic first-pass metabolism and diminishing presystemic degradation [62]. This lipid-digesting substance stimulates the gallbladder to secrete endogenous biliary lipids, such as cholesterol, phospholipids, and bile salts. By forming mixed micellar structures and micelles, these secretions improve the solubilization and absorption of CoQ. The micelles' polar head group penetrates the aqueous phase, while the nonpolar hydrocarbon tail group extends into the core [63, 64]. Furthermore, incorporating TPGS and lecithin in the formulation as amphiphilic substances may have facilitated the drug molecule's bypassing of hydrophobic interactions with the unstirred mucus layer, enhancing drug absorption [65]. The extended circulation time and enhanced bioavailability of CoQ in the SLN formulation indicate a more significant potential for crossing the BBB and entering the CNS, offering it a promising approach for neuroprotective applications. The results emphasize the potential of lipid-based nanocarriers in improving drug delivery, decreasing the dose frequency, and maximizing the pharmacological effects of bioactive substances such as CoQ for therapeutic applications.

### 3.5. Brain Distribution of CoQ Samples

**Figure 6:**
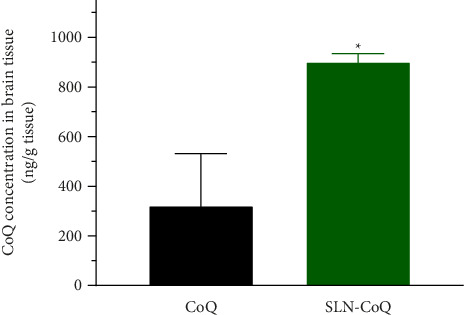
Brain tissue distribution of CoQ following the oral administration of CoQ samples (100 mg CoQ/kg). Data represent mean ± S.E. of four experiments.

The brain tissue distribution of CoQ in rats was significantly enhanced when delivered through SLN-CoQ compared to that of crystalline CoQ (Figure 6). The mean concentration of crystalline CoQ in brain tissue was 322.28 ± 296.8 ng/g. In contrast, the mean concentration of SLN-CoQ reached 901.44 ± 33.04 ng/g, indicating a nearly 3-fold increase in bioavailability. This improvement can be attributed to the SLNs' ability to enhance the solubility and stability of CoQ, promoting better absorption and transport across the BBB. The lipid matrix of SLNs enables a prolonged release of CoQ, enhancing its systemic availability and facilitating its entry into the brain. Furthermore, the nanosized particles of SLNs can use the endothelial cells of the BBB through mechanisms such as endocytosis, hence facilitating increased brain uptake [66]. This approach of employing SLN-CoQ may address the limited bioavailability and inadequate BBB permeability often associated with free CoQ formulations. The observed increase in brain tissue levels of CoQ may enhance treatment effectiveness in managing neurodegenerative disorders, where CoQ is recognized for its advantageous properties. The findings indicate that SLNs may serve as a promising strategy for CoQ, improving its biopharmaceutical characteristics and providing a more efficient pathway to the brain for neuroprotective applications.

## 4. Conclusion

The strategic application of SLN-CoQ significantly improves the biopharmaceutical characteristics of CoQ, enhancing its solubility, colloidal stability, and bioavailability. Pharmacokinetic investigations in rats revealed a marked enhancement in the systemic exposure of CoQ when administered as SLNs compared to that of crystalline CoQ. The SLN-CoQ formulation exhibited an almost 3-fold increase in brain tissue concentration, demonstrating the improved bioavailability and ability of SLNs to promote CoQ's transport across the BBB. The findings indicate that SLNs offer an attractive approach for enhancing CoQ transport to the brain, potentially improving therapeutic outcomes for neurological disorders. However, future research needs to investigate long-term stability, in vivo efficacy, and clinical translation to fully leverage the potential of SLN-CoQ in therapeutic applications targeting the brain.

## Figures and Tables

**Table 1:** Optimization of a suitable ratio of the polymers.

	Ratio SA:TPGS:SL:CoQ	EE (%)	DL (%)	% dissolved at 120 min	Initial dissolution rate (hr^−1^)	Diffusion through AM (μg/mL)
Crystalline CoQ				1.1 ± 0.14	0.0038 ± 0.002	0.13 ± 0.056
F1	40:20:5:1	62.36 ± 6.41	1.71 ± 0.13	74.7 ± 1.74	0.1820 ± 0.122	2.34 ± 0.268
F2	40:20:10:1	85.22 ± 5.00	2.20 ± 0.11	99.9 ± 3.66	0.5095 ± 0.129	5.43 ± 0.289
F3	40:15:5:1	83.88 ± 6.44	2.17 ± 0.14	67.7 ± 11.46	0.3850 ± 0.101	3.03 ± 0.121
F4	40:15:10:1	81.58 ± 3.94	2.12 ± 0.08	15.8 ± 10.24	0.0582 ± 0.056	3.88 ± 0.552
F5	40:10:5:1	82.42 ± 6.37	2.13 ± 0.13	33.9 ± 16.23	0.1587 ± 0.040	1.77 ± 0.513
F6	40:10:10:1	77.40 ± 4.55	2.03 ± 0.10	31.0 ± 6.06	0.1121 ± 0.057	2.72 ± 0.675
Ref				3.3 ± 0.21	0.0310 ± 0.002	

*Note:* CoQ, coenzyme Q10; TPGS, α-tocopheryl polyethylene glycol 1000 succinate.

Abbreviations: AM = artificial mucus, DL = drug loading, EE = entrapment efficiency, SA = stearic acid, and SL = soy lecithin.

**Table 2:** Pharmacokinetic parameters of CoQ samples following oral administration to rats.

	Crystalline CoQ (100 mg/kg; p.o.)	SLN-CoQ (100 mg-CoQ/kg; p.o.)
*C*_max_ (ng/mL)	69.15 ± 17.27	308.48 ± 49.25
*T*_max_ (h)	1.67 ± 2.02	3.33 ± 1.15
AUC_0–6_ (ng·h/mL)	488.1 ± 89.7	1002.5 ± 122.3
AUC_0–∞_ (ng·h/mL)	1178.19 ± 498.85	4203.75 ± 2189.11
MRT (h)	23.15 ± 9.49	35.27 ± 25.58
*t*_1/2_ (h)	15.34 ± 6.27	30.28 ± 20.64
*K*_*e*_ (h^−1^)	0.049 ± 0.01	0.046 ± 0.05

*Note:C*_max_: maximum concentration; *T*_max_: time to maximum concentration; AUC_0–∞_: area under the curve of blood concentration *vs*. time from 0 h to infinity; *t*_1/2_: elimination half-life; and *K*_*e*_: elimination rate constant. Data represent mean ± SE of four experiments.

Abbreviation: MRT = mean residence time.

## Data Availability

This manuscript has all the data that support the study's results.
